# The Columbia-Bronx VA amalgamative clerkship: an effective, 12-week, integrated, longitudinal clinical experience

**DOI:** 10.1080/10872981.2017.1301630

**Published:** 2017-03-20

**Authors:** Jillian Diuguid-Gerber, Samuel Porter, Samuel C. Quiah, Katherine Nickerson, Deborah Jones, Zeena Audi, Boyd F. Richards

**Affiliations:** ^a^ PGY1 in Internal Medicine, New York University Langone Medical Center, New York, NY, USA; ^b^ PGY1 in Internal Medicine, University of Colorado-Denver Medical Center, Denver, CO, USA; ^c^ Center for Education Research and Evaluation, Columbia University Medical Center, New York, NY, USA; ^d^ Department of Medicine, Columbia University College of Physicians and Surgeons, New York, NY, USA; ^e^ PGY3 in Pediatrics, Boston Children’s Hospital, Boston, MA, USA; ^f^ Medical Education Research and Scholarship, University of Utah School of Medicine, Salt Lake City, UT, USA

**Keywords:** Student, education, urban, veterans, patient, continuity, systems, career

## Abstract

**Background:** Many medical schools have adopted the longitudinal integrated clerkship (LIC) model in response to calls for increased continuity in clinical learning environments. However, because of implementation challenges, such programs are not feasible at some institutions or are limited to a small number of students.

**Objective:** In January 2014, Columbia University College of Physicians and Surgeons (P&S) recognized the need to explore different LIC formats and began offering four, 12-week amalgamative clerkships (AC). Students within this curricular track experienced primary care, internal medicine ‘away’, orthopedic surgery, urology, and an elective in an integrated format.

**Design:** P&S developed the AC in partnership with the James J. Peters VA Medical Center in Bronx, NY (BVA). All patient care and educational conferences took place at the BVA during the 12-week experience. The learning objectives of the AC were aligned to the learning objectives of a 52-week LIC also offered at Columbia. An evaluation process was developed to determine student learning experiences and preliminary outcomes, including how well the LIC-related objectives could be achieved in a shorter period of time.

**Results:** In 2015, P&S collected AC evaluation data through three student feedback sessions. Students reported that the AC provided opportunity for patient continuity, patient-centered care approaches, meaningful roles for students, career development opportunities, and health systems awareness.

**Conclusions:** Early outcomes indicate that the BVA AC provides a degree of longitudinality that can influence student perceptions of patient care, career development, and health systems, consistent with the larger LIC. The team continues to gather additional data on students’ experiences and investigate additional sites that have potential to serve as future AC learning environments.

## Introduction

Many medical schools have adopted a comprehensive longitudinal integrated clerkship (LIC) model in response to calls for increased continuity in clinical learning environments.[1,2] This model is an alternative to block rotations, which traditionally comprise the third year of medical school. The LIC typically spans a full year and features three common elements: medical student participation in comprehensive patient care over extended periods of time, continuity of learner–preceptor relationships, and a multidisciplinary, integrated approach to clinical education in required core clerkships.[3] Students learn complexities of disease from multiple perspectives, and develop deeper understanding of health systems. However, because of logistical implementation challenges,[4] such programs are not feasible at some institutions or are limited to a small number of students.

While Columbia University College of Physicians and Surgeons (P&S) offers a year-long rural LIC for 10 students a year at Bassett Medical Center in Cooperstown, NY, USA,[5] the school recognized the need to explore alternative integrated clerkship formats more feasible in the setting of a large teaching hospital in an urban setting. As a result, in 2013, P&S introduced an abbreviated, 12-week, amalgamative clerkship (AC), in partnership with the James J. Peters VA Medical Center in Bronx, NY, USA (BVA). The purpose of this pilot program was to provide opportunities for students to experience continuity in patient care at an urban medical center and to begin to evaluate the extent to which learning outcomes of 52-week LICs could be achieved in a shorter period of time, thereby increasing the number of students experiencing the benefits of an integrated, longitudinal format.

## Intervention

In January 2014, P&S began offering four 12-week AC sessions per year, each with four students. The AC was developed by faculty from the Department of Medicine and the Center for Education Research and Evaluation at Columbia University in collaboration with faculty from the BVA. Students volunteered for the 16 slots, with random selection used to fill any remaining vacancies.

The BVA serves an urban population of 24,000 veterans, active duty military personnel, and their families, with high disease complexity and psychosocial stress. Applying the patient-centered medical home model, BVA care is organized around Patient Aligned Care Teams (PACTs) which include physicians, registered nurses, medical assistants, pharmacists, psychologists, social workers, and dieticians serving one patient panel.

### Educational objectives the amalgamative clerkship experience

Previous studies have shown that several learning objectives are enhanced when students experience continuity in clinical learning environments, including creating opportunities for continuity with patients and preceptors,[4] promoting patient-centered values and approaches,[6] creating significant roles for medical students,[7] supporting professional identity formation,[8,9] and increasing health systems awareness.[10] The purpose of our program evaluation was to explore students’ perceptions of how well the 12-week format helped them achieve these learning outcomes in a 12-week period of time.

The AC integrates five of the standard 10 clerkships of the P&S Major Clinical Year (MCY). During a 12-week period when most students in a cohort of 30–40 take traditional block clerkships in primary care (five weeks), orthopedic surgery (one week), urology (one week), electives (one week), and a rotation in medicine at an affiliate hospital (four weeks), four students experience these rotations in the AC format. These four students experience all other rotations during their MCY (medicine at the home institution, surgery, neurology, psychiatry, pediatrics, and obstetrics and gynecology) in the traditional block rotation format.

**Figure 1.**
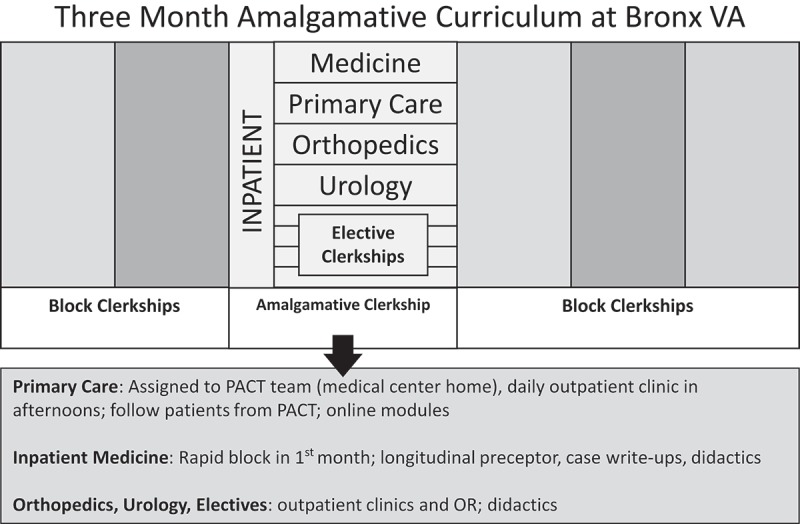
Three Month Amalgamative Curriculum at Bronx VA.

In this way, AC students partake in a hybrid curriculum during their MCY, with 12 weeks devoted to the AC and 38 weeks to block clerkships (see Figure 1). In the AC, students participate in integrated, collaborative patient care as they travel with their self-created panel of patients throughout inpatient, outpatient, and surgical settings while still fulfilling all of the traditional objectives of these rotations.

## Context

### Program structure: amalgamative clerkships

The BVA AC begins with a three-week inpatient immersion experience. The remaining nine weeks of the AC experience are spent in integrated clerkships. In the integrated clerkship period, students see patients independently under preceptor supervision in a variety of settings.

Students typically spend four to five afternoons per week in primary care clinic with an assigned PACT, one to two half days per week in orthopedics, one to two half days per week in urology, and one to two half days per week in an elective (such as Emergency Medicine, Radiation Oncology, Physical Medicine and Rehabilitation, Spinal Cord Injury, Dermatology, and Infectious Disease).

Urology and Orthopedics take place in both the operating room and outpatient clinic settings. Students assist in procedures and surgeries. Longitudinally, students have the opportunity to see patients before, at and during the recovery period from their surgeries.

The AC features many didactic opportunities in addition to the experiential learning in the inpatient and outpatient settings. BVA physicians lead case-based discussions addressing common and important chief complaints. Student-driven weekly preceptor sessions involve case and topic presentations and bedside teaching. Students complete web modules on key primary care subjects, such as diabetes and depression. Clerkship directors meet with students throughout the 12 weeks to discuss longitudinal patients, providing opportunity to understand development of pathophysiology over time, as well as process emotions that arise as students form connections with patients.

In May, June, and September of 2015, we collected student perception data from students who completed the AC in 2014. Eligible students were invited to participate in one of three voluntary student feedback sessions, through an email invitation from J.D.G. A total of 16 students participated. The authors developed a feedback session interview guide, which focused on learning from patient care experiences during their AC and block rotations. AC students experienced both the AC and block rotations, so they were able to compare their perceptions of both learning outcomes. The feedback sessions were led by S.Q. and B.F.R. and audiotaped and transcribed verbatim. All identifying information was removed and the authors met several times to analyze and draw preliminary conclusions from the feedback session data.

## Outcomes

We present a summary of comments from AC students as they pertain to each stated AC educational objective (see Table 1 for exemplar quotes).

**Table 1.** Educational objectives with representative quotes from AC students who participated in the feedback sessions.

Objectives	Representative student quotes
Patient continuity	‘My first patient at BVA came in for drug use and we found cancer, he was discharged and I lost track of him for a month. After a month he runs up to me in the hall on the way to chemo session and I was able to visit him weekly.’
	‘One patient I was with during recovery from alcohol abuse. He was committed, recovered and it allowed him to get needed surgery. It was nice to see doctors along the way to his treatment, being involved in surgery and following up with him after.’
Patient-centeredness	‘Say you saw a patient and probably have seen them in every single clinic the VA has, you can say, okay, hey doc, even though patient expressed this, they had this family situation going on, so that plan, let’s think of something new.’
	‘You saw people get better, things in people’s lives change … you have urinary retention, we fixed it, now you are able to go on vacation with your grandchildren.’
	‘You had to keep up with patients and you had stewardship over patients … at BVA you would run into your clients in the hallway.’
Creating meaningful roles	‘… you’re learning just as much working on motivational interviewing. We were making choices that were more valuable to me than to be asked a few more times what the A1C levels are’
	‘A patient was really sick and I got to know his wife because he was in a ton of pain and he kept getting readmitted … the wife wasn’t getting along with team well and would only talk to me so I was the liaison between patient and team.’
	‘It just made me feel like I was actually useful.’
Career Development	‘His breathing could be heard from the hallway—biphasic stridor … The man had laryngeal cancer and had difficulty breathing over the last week … I relayed the pertinent history, and, within minutes, the patient underwent flexible laryngoscopy revealing a 2-mm airway between fixed vocal cords. Half an hour later, I was scrubbed in on the patient’s tracheotomy … I contemplated the swift, decisive, and life-preserving actions taken by the head & neck surgeons. I knew in that moment that I was interested in a career in Otolaryngology.’
	‘It lets you think about what you’re interested in, and attach to that. For me, substance abuse was something that I was really interested in, and I definitely gravitated towards patients who were having problems with substance abuse and got to follow them long-term.’
	‘There was just so much autonomy, so, like, you formed your own attitudes [about] how you wanted to practice as a physician.’
Health systems awareness	‘I appreciated the integrated system so you could see everything pertinent to a patient in one place’
	‘When you experience frustrations with the patient as they go through it, it makes you … understand the flaws of the system more, and blame individual people within the system less’
	‘USPSTF recommendations are built into the system and there is an emphasis on [evidence based practice] so there aren’t as many instances of unnecessary treatments like MRI.’

### Patient continuity

Students commented that while the block clerkships do provide some opportunities for students to learn about continuity of care, there are more deliberate opportunities to learn about continuity in the AC. Students longitudinally worked with patients in the hospital, in skilled nursing facilities, and in their homes. They followed their hospitalized patients post-discharge and saw patients in outpatient specialty clinics, reporting back to patients’ primary care physicians.

AC students experienced multiple transitions in care, allowing them to see how patients’ diagnoses evolved, adding dimensions of experience not available to students in traditional block rotations. Students contrasted different perspectives and approaches to treatment as patients were treated by different specialists, who were solving different problems for the patient or solving the same problem in different ways.

### Patient-centeredness

AC students developed meaningful relationships with their longitudinal patients over the 12-week experience. They reported that the emphasis in the AC is on ‘what you should be doing for your patient,’ rather than strictly attaining academic knowledge.

Students immersed themselves in the narratives of VA patients’ lives. As relationships developed, students learned patients’ stories, putting their complex cases into perspective. Students began to understand the lifestyles, hopes, and values of their patients. The likelihood of experiencing success with patients was enhanced because students genuinely had a chance to get to know their patients as people and advocate for them where they needed it most.

Students felt that many physicians and patients knew each other due to the small community feel, and felt this contributed to patient-centered care. As key members of this community, students valued being patient advocates and liaisons between patients and healthcare teams. Students also felt they made their patients’ health care experience less alienating by being a supportive, repeated presence.

### Creating significant roles for students

AC students felt encouraged to know their patients better than any of the other health care providers at the VA. The longitudinal, integrated structure of being part of a PACT gave these students the chance to take active roles in their medical teams and in advancing their patients’ care. They practiced motivational interviewing and counseled their patients in behavioral change over time, appreciating the effects of their efforts in patients’ smoking cessation and abstinence from alcohol.

Students advocated for patients within different clinical settings, and confidently collaborated with patients and providers. Integration, collaboration, and familiarity with the system all contributed to the creation of more ‘doctor-like’ roles for students, which have been associated with student confidence and satisfaction with their clerkships.[7]

### Career development

AC students focused on following patients being cared for by their specialty of interest and, due to their level of autonomy in selecting their patient panels, were able to develop their career interests in the AC more than when they were in the block curriculum.

### Health systems awareness

AC students valued the diversity of experience with different patient populations and approaches to patient care made available to them through the 12-week integrated format. Furthermore, the PACTs exposed students to a multidisciplinary model of care, through which students learned how interrelated roles in a team function together around a single patient’s care.

Students also leveraged the VA EMR system, which provided rich patient information going back many years. Each AC student undertook a quality improvement project during their 12 weeks, culminating with a final presentation. Many of these projects were data-driven, utilizing the robust EMR data capacity. This differs from the traditional primary care clerkship, where students give a topic presentation but are not asked to complete a quality improvement project.

## Discussion

We find that students who participate in the Bronx VA AC program benefit from longitudinal patient contact, patient-centeredness, meaningful roles, and health systems awareness, and opportunity for professional identity development.

These outcomes encourage us to conclude that students who participated in the AC met the program’s educational objectives. As we continue to gather additional data on students’ experiences, we hope to evaluate the impact of this program and compare its effect on students to that of the Columbia year-long LIC at Bassett in Cooperstown, NC, USA. More qualitative interviews and focus groups are forthcoming, as are multi-year aggregate data from a survey given to students at the end of the MCY.

Other medical schools may benefit from setting up an AC, specifically in partnership with a VA. We believe a VA hospital is an ideal site for such an undertaking, given the opportunities for continuity between inpatient, outpatient and rehabilitative settings, and the strength of the VA healthcare system’s primary care model. Other sites with similar profiles have potential to serve as an AC setting, and we plan to investigate expanding to these sites in the future.
